# Triglyceride-glucose index and prognosis in non-diabetic critically ill patients: data from the eICU database

**DOI:** 10.3389/fmed.2025.1558968

**Published:** 2025-04-08

**Authors:** Xi Li, Qiujin Lin, Dewen Zhang, Zhenhua Huang, Jinshi Yu, Jiaqi Zhao, Wenzhou Li, Wei Liu

**Affiliations:** ^1^Pharmacy Department, Shenzhen Qianhai Shekou Free Trade Zone Hospital, Shenzhen, China; ^2^Department of Critical Care Medicine, Pengpai Memorial Hospital, Shanwei, China; ^3^Department of Pharmacy, Pengpai Memorial Hospital, Shanwei, China; ^4^Department of Emergency Medicine, Health Science Center, Shenzhen Second People’s Hospital, The First Affiliated Hospital of Shenzhen University, Shenzhen, China; ^5^Shenzhen Baoan Women’s and Children’s Hospital, Shenzhen, China; ^6^Department of Emergency Medicine, The Huangpu People’s Hospital, Zhongshan, China

**Keywords:** triglyceride-glucose index, insulin resistance, intensive care unit, non-diabetes, all-cause mortality, nonlinear correlation

## Abstract

**Background:**

The triglyceride-glucose (TyG) index is a marker for insulin resistance (IR) linked to diabetes complications and poor outcomes. Its connection to all-cause mortality in non-diabetic critically ill patients is unknown. This study aims to investigate the TyG index’s impact on mortality in this population, evaluating how IR affects their prognosis.

**Methods:**

This study is retrospective observational research utilizing data from the eICU Collaborative Research Database. A total of 14,089 non-diabetic critically ill patients were included and categorized into three groups based on the TyG index measured on the first day of admission (T1, T2, and T3). Kaplan-Meier survival analysis was performed to compare the 28-day mortality rates among the different groups. Cox proportional hazards models were used to assess the relationship between the TyG index and 28-day mortality. Additionally, we conducted sensitivity analyses, subgroup analyses, and interaction analyses to assess the robustness of the results.

**Results:**

During the observation period, 730 patients (5.18%) died in the ICU, while 1,178 patients (8.36%) died in the hospital. The 28-day ICU mortality rate and hospital mortality rate significantly increased with higher TyG index values (*P* < 0.001). Cox proportional hazards models were used to assess the relationship between the TyG index and 28-day mortality. Specifically, Cox proportional hazards models were used to assess the relationship between the TyG index and 28-day mortality. Furthermore, the analysis showed a nonlinear effect of the TyG index on mortality in non-diabetic critically ill patients, with a critical point at 9.94. While Below 9.94, ICU and hospital mortality rates rose with higher TyG index values. But above 9.94, mortality didn’t significantly increase despite further rises in the TyG index. Sensitivity and subgroup analyses confirmed the robustness of these results, and E-value analysis indicated strong resistance to unmeasured confounding factors.

**Conclusion:**

The TyG index demonstrates a significant positive correlation with all-cause mortality in non-diabetic critically ill patients, exhibiting a nonlinear relationship. Consequently, the TyG index serves as a crucial tool for identifying high-risk patients, thereby assisting clinicians in formulating more effective monitoring and intervention strategies.

## 1 Introduction

The mortality rate among critically ill patients in the ICU is alarmingly high, particularly in cases of sepsis, respiratory failure, and multiple organ dysfunction syndrome (1–4). Therefore, timely risk prediction and intervention could significantly reduce mortality rates. Common tools for assessing the prognosis of critically ill patients include the Acute Physiology and Chronic Health Evaluation II (APACHE II), the Simplified Acute Physiology Score II (SAPS II), and the Sequential Organ Failure Assessment (SOFA) (5, 6). However, these existing scoring tools are not without limitations and cannot be effectively utilized in the absence of essential clinical information. Furthermore, traditional tools often inadequately assess metabolic disorders in patients, particularly in evaluating the impact of metabolic issues such as IR on the prognosis of critically ill patients. Consequently, there is an urgent need to identify new biomarkers that can serve as alternatives or supplements to enhance prognosis assessment and treatment strategies for critically ill patients.

Insulin resistance is defined as the decreased sensitivity of peripheral tissues to insulin (7), and it plays a critical role in many metabolic abnormalities associated with critical illness, being linked to increased morbidity and mortality (8). In recent years, the TyG index has garnered attention as a simple and accessible biomarker. The TyG index is calculated using fasting blood glucose and triglyceride levels, and it has been shown to correlate closely with IR (9), demonstrating good predictive value across various clinical settings (10–12). Studies have indicated a significant relationship between the TyG index and the risk of death in critically ill patients suffering from conditions such as sepsis, intracerebral hemorrhage, coronary heart disease, and atrial fibrillation, thereby providing new insights for risk prediction in this vulnerable population (13–16).

In critically ill patients, the proportion of non-diabetic individuals is significantly higher than that of diabetic patients (17, 18). A large retrospective analysis revealed that the association between mortality risk and hyperglycemia in non-diabetic critically ill patients is markedly stronger than in diabetic patients. This may be attributed to the higher prevalence of hyperglycemia among non-diabetic critically ill patients and the more pronounced impact of hyperglycemia on their prognosis (19–21). This finding underscores the necessity of focusing on the risks faced by critically ill patients who have not been diagnosed with diabetes. Previous studies have shown that the triglyceride to fasting insulin ratio (TyG index) is an effective predictor of future ischemic heart disease risk in non-diabetic individuals (22). This suggests that the TyG index could serve as a significant indicator for assessing cardiovascular event risk in non-diabetic adults, thereby holding clinical application value. This conclusion lays a solid foundation for understanding the clinical relevance of the TyG index across different populations. Although existing studies have mainly concentrated on diabetic patients or the general population, research specifically targeting non-diabetic critically ill patients remains limited.

To our knowledge, one study indicated that the predictive value of IR assessed via the TyG index appears to be more significant in patients without diabetes (DM). However, this study’s small sample size may render the results more susceptible to outliers, thereby compromising their stability. Therefore, further exploration of the potential application of the TyG index in non-diabetic critically ill patients will not only enhance our understanding of this indicator but also aid in developing more targeted treatment plans to improve the prognosis of this patient population.

## 2 Materials and methods

### 2.1 Study design

This study is a retrospective observational research project that utilizes data from the international online database known as the eICU Collaborative Research Database (eICU-CRD). This database was established through a collaboration between Philips and the Massachusetts Institute of Technology (MIT) Laboratory for Computational Physiology (LCP), encompassing clinical data from over 200,000 patients collected across 335 ICU units in 208 hospitals throughout the United States between 2014 and 2015. The data is widely employed in various observational studies, addressing aspects such as patients’ vital signs, severity of illness assessments, diagnostic information, and outcome variables. To protect patient privacy, all data has been anonymized and is fully compliant with the Health Insurance Portability and Accountability Act (HIPAA) regulations. Data access was granted following the completion of the Collaborative Institutional Training Initiative (CITI) program’s “Data or Sample Only Research” course, which exempted the study from review by the MIT Institutional Review Board (Record ID: 49995491), and informed consent was not required. This study strictly adhered to the Declaration of Helsinki, ensuring that all research methods complied with relevant ethical standards and guidelines.

### 2.2 Population

**FIGURE 1:**
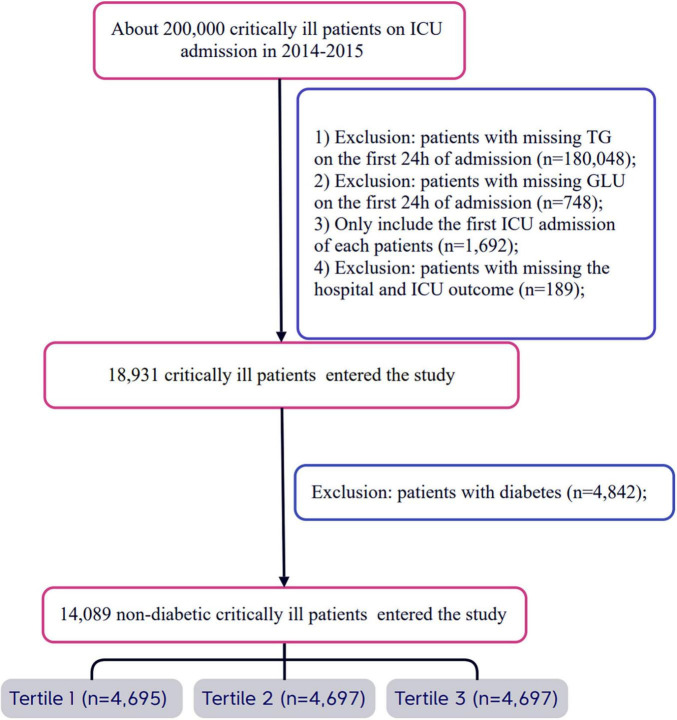
Flow chart of study population. ICU, intensive care unit.

All eligible patients were diagnosed using ICD-9 codes from the eICU Collaborative Research Database. Strict exclusion criteria were established during the patient selection process. First, patients who did not provide triglyceride and glucose data on the day of admission were excluded. Second, for patients with multiple admissions, only data from their first ICU admission were analyzed, while records from subsequent admissions were excluded. Additionally, patients lacking hospital and ICU outcome information were also excluded. Finally, any patients with a prior diagnosis of diabetes or those diagnosed with diabetes upon admission were not included in the study. After applying these stringent selection criteria, a total of 14,089 patients were included and categorized into three groups based on the tertiles of the TyG index measured on the first day of admission. For a detailed overview of the patient selection process, please refer to Figure 1.

### 2.3 Variable extraction

The eICU database encompasses a range of critical medical records, including demographic information, physiological parameters obtained from bedside monitoring devices, and disease diagnoses coded according to the International Classification of Diseases, Ninth Revision, Clinical Modification (ICD-9-CM). Furthermore, the database incorporates additional laboratory data collected during standard medical procedures. Relevant data for all participants were extracted from the eICU-CRD system within the first 24 h following admission. The physiological variables collected primarily include temperature (°C), respiratory rate, heart rate (HR), and mean arterial pressure (MAP). Baseline characteristics such as age, sex, race, and weight were gathered from patient information forms. Laboratory indicators related to total cholesterol, triglycerides, high-density lipoprotein cholesterol (HDLc), and low-density lipoprotein cholesterol (LDLc) were obtained from laboratory information forms. The severity of illness at the time of admission was evaluated using the SOFA scoring system.

### 2.4 Definition and main results of the TyG index

The TyG index is calculated using fasting blood glucose and triglyceride levels at the time of admission, employing the formula: TyG index = ln[fasting triglycerides (mg/dL) × fasting glucose (mg/dL) / 2] (23, 24). The primary outcome of this study is the all-cause mortality rate within 28 days of hospitalization, encompassing inpatient deaths that occur in both the ICU and general ward.

### 2.5 Statistical analysis

Continuous variables that follow a normal distribution are typically expressed as mean ± standard deviation (SD), whereas non-normally distributed continuous variables are described by the median and inter-quartile range (IQR). Categorical data are usually presented as counts and percentages. Comparisons between continuous variables based on the tertiles of the TyG index were conducted using the Student’s *t*-test, while categorical variables were analyzed with Pearson’s chi-square test or Fisher’s exact test. Additionally, we applied Cox proportional hazards models to assess the association between the TyG index and 28-day mortality, presenting results as hazard ratios (HR) with 95% confidence intervals (CI). These results include both crude regression estimates and estimates adjusted for covariates. We selected adjusted covariates based on the association between confounding factors and the outcomes of interest, using a criterion of greater than 10% change in effect estimates. In terms of clinical significance, we considered and adjusted for the following covariates: sex, age, race, body mass index (BMI), SOFA score, blood urea nitrogen (BUN), serum calcium, total cholesterol (TC), high-density lipoprotein (HDL), red blood cell count (RBC), hemoglobin (HGB), white blood cell count (WBC), red cell distribution width (RDW), liver failure, immunosuppression, and cirrhosis. Furthermore, we utilized restricted cubic spline (RCS) regression models with three knots to characterize the relationship between the TyG index and the hazard ratio. We then applied a piecewise linear regression model to further investigate the saturation effect of the TyG index on mortality. Additionally, we performed likelihood ratio tests and conducted comparative analyses between the single-segment linear regression model and the piecewise linear regression model. To calculate the 95% confidence interval for the turning point, we employed the bootstrap resampling method, as described in previous analyses. To ensure the robustness of the results, we conducted sensitivity analyses. The calculation of E-values indicated the strength of unmeasured confounding variables required to nullify the observed effect (25, 26). All statistical analyses were performed using EmpowerStats (X&Y Solutions, Inc., Boston, MA, United States)^1^ and R software version 3.6.1^2^, with a two-sided α level set at 0.05.

## 3 Results

### 3.1 Baseline characteristics of the included participants

A total of 14,089 patients met the inclusion criteria in the eICU database (Figure 1). The average age of the patients was 63.47 ± 15.89 years, with approximately 41.78% of the patients being male (Supplementary Table 1). The baseline TyG index values ranged from 5.70 to 14.44, with a mean of 8.83 (Supplementary Figure 1). Among these critically ill patients, 730 (5.18%) died in the ICU within 28 days, and a total of 1,178 (8.36%) patients died in the hospital.

We presented that the baseline characteristics of the patients grouped by the tertiles of the TyG index (Table 1). The significant differences were observed in multiple health parameters as the TyG index increased from T1 to T3. The average age of participants in the T3 group was 59.74 years, compared to 66.45 years in the T1 group (*P* < 0.001), indicating that participants in the T3 group were younger. Additionally, BMI significantly increased with the higher tertiles of the TyG index. Relevant laboratory results also showed significant differences: the SOFA score, BUN, glucose, total cholesterol, triglycerides, WBC count, RBC count, and HBG all significantly increased with the TyG index (*P* < 0.001). Differences in sex and race among the three groups were also noted. Furthermore, both ICU and hospital lengths of stay were significantly prolonged as the TyG index increased (*P* < 0.001).

**TABLE 1:** The baseline characteristics of participants according to tertiles of TyG index.

Characteristics	T1(5.70–8.49)	T2(8.49–9.04)	T3(9.04–14.44)	*P*-value
Age (year)	66.45 ± 16.56	64.23 ± 15.35	59.74 ± 14.97	<0.001
BMI (kg/m^2^)	26.83 ± 6.90	28.90 ± 7.54	30.58 ± 7.75	<0.001
**Gender**				0.005
Male, *n* (%)	2,034 (43.32%)	1,971 (41.97%)	1,880 (40.03%)	
Female, *n* (%)	2,661 (56.68%)	2,725 (58.03%)	2,816 (59.97%)	
**Ethnicity**				<0.001
Caucasian, *n* (%)	3470 (74.53%)	3607 (77.75%)	3571 (76.89%)	
African-American, *n* (%)	688 (14.78%)	532 (11.47%)	482 (10.38%)	
Hispanic, *n* (%)	253 (5.43%)	245 (5.28%)	295 (6.35%)	
Asian, *n* (%)	151 (3.24%)	168 (3.62%)	190 (4.09%)	
Native American, *n* (%)	22 (0.47%)	17 (0.37%)	26 (0.56%)	
Unknown, *n* (%)	72 (1.55%)	70 (1.51%)	80 (1.72%)	
SOFA score, median (IQR)	1.00 (1.00**–**3.00)	1.00 (0.00**–**4.00)	1.00 (0.00**–**4.00)	<0.001
**Laboratory tests**				
BUN, median (IQR), (mmol/L)	16.00 (11.00**–**23.00)	16.00 (12.00**–**23.00)	17.00 (12.00**–**26.00)	<0.001
Serum calcium	8.48 ± 0.70	8.50 ± 0.71	8.44 ± 0.81	<0.001
GLU, median (IQR), (mg/dl)	104.00 (92.00**–**121.00)	117.00 (101.00**–**139.00)	141.00 (114.00**–**189.00)	<0.001
HGB, (g/L)	11.97 ± 2.13	12.29 ± 2.31	12.46 ± 2.35	<0.001
PLT, (×10^9^/L)	205.87 ± 87.42	213.95 ± 85.15	217.48 ± 84.65	0.715
TC, (mg/dl)	141.21 ± 40.64	155.92 ± 44.59	173.20 ± 57.18	<0.001
TG, median (IQR), (mg/dl)	66.00 (53.00**–**80.00)	107.00 (89.00**–**127.00)	183.00 (141.00**–**244.00)	<0.001
HDL, (mg/dl)	49.26 ± 18.04	42.03 ± 14.70	36.02 ± 13.46	<0.001
RBC, (×10/^12^L)	3.98 ± 0.70	4.11 ± 0.74	4.15 ± 0.76	<0.001
RDW, (%)	14.64 ± 2.18	14.59 ± 2.18	14.48 ± 2.02	0.003
WBC, (×10^9^/L)	9.96 ± 5.31	11.14 ± 5.79	12.69 ± 8.75	<0.001
**Comorbidities**				
Hepatic failure, *n* (%)	40 (0.92%)	30 (0.69%)	21 (0.50%)	0.067
Lymphoma, *n* (%)	5 (0.11%)	12 (0.27%)	11 (0.26%)	0.205
Metastatic cancer, *n* (%)	49 (1.12%)	64 (1.47%)	54 (1.28%)	0.370
Leukemia, *n* (%)	13 (0.30%)	25 (0.57%)	29 (0.69%)	0.035
Immunosuppression, *n* (%)	60 (1.38%)	81 (1.85%)	95 (2.26%)	0.01
Cirrhosis, *n* (%)	52 (1.19%)	35 (0.80%)	27 (0.64%)	0.019
COPD, *n* (%)	227 (4.83%)	227 (4.83%)	191 (4.07%)	0.121
CRF, *n* (%)	358 (7.63%)	341 (7.26%)	257 (5.47%)	<0.001
AMI, *n* (%)	751 (16.00%)	971 (20.67%)	1045 (22.25%)	<0.001
**Outcomes**				
**28-day ICU mortality**				<0.001
No, *n* (%)	4535 (96.59%)	4469 (95.15%)	4355 (92.72%)	
Yes, *n* (%)	160 (3.41%)	228 (4.85%)	342 (7.28%)	
**28-day hospital mortality**				<0.001
No, n (%)	4403 (93.78%)	4318 (91.93%)	4190 (89.21%)	
Yes, n (%)	292 (6.22%)	379 (8.07%)	(10.79%)	
ICU LOS, d	2.78 ± 3.63	3.09 ± 4.20	3.49 ± 4.65	<0.001
Hospital LOS, d	6.53 ± 7.83	7.04 ± 9.89	7.45 ± 11.37	<0.001

Continuous variables are summarized as mean (SD) or median (trisection interval); categorical variables are presented as percentages (%). BMI, body mass index; COPD, chronic obstructive pulmonary disease; AMI, acute myocardial infarction; ICU, intensive care unit; LOS, length of stay; BUN, blood urea nitrogen; RBC, red blood cell; HGB, hemoglobin; PLT, platelets; SOFA, sequential organ failure assessment; GLU, glucose; TC, total cholesterol; TG, triglycerides; HDL, high-density lipoprotein; RDW, red cell distribution width; WBC, white blood cells; CRF, chronic renal failure.

**FIGURE 2:**
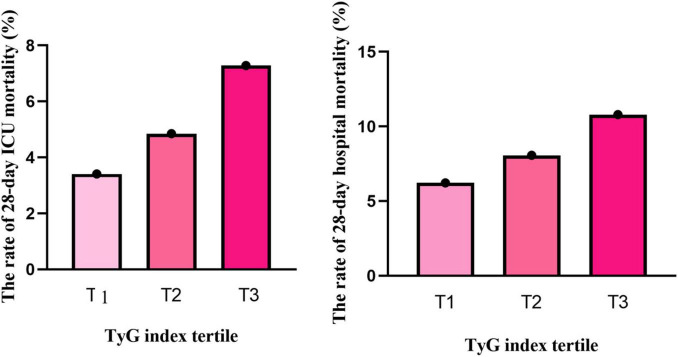
The relationship between TyG index tertiles and the rate of 28-day mortality. Both ICU mortality and hospital mortality rates increased with elevated TyG index (*p <* 0.001).

Mortality analysis revealed a concerning trend: the 28-day mortality rate in the ICU increased from 3.41% in T1 to 7.28% in T3 (*P* < 0.001), and the hospital mortality rate also rose from 6.22% to 10.79% (*P* < 0.001), highlighting the potential of the TyG index as a prognostic biomarker (Figure 2).

### 3.2 Incidence rate of all-cause mortality among different groups

The 28-day cumulative mortality rates for patients in the ICU and the hospital were 5.18% and 8.36%, respectively (Table 1). As the tertiles of the TyG index increased, both the 28-day ICU mortality rate and hospital mortality rate exhibited a significant upward trend (trend test *P* < 0.001). In the study, we used the Kaplan–Meier curves for 28-day ICU and hospital mortality (Supplementary Figure 2). As the tertiles of the TyG index increased, the probability of 28-day ICU and hospital mortality progressively rose, indicating that patients with the highest TyG index had an elevated likelihood of dying in the ICU and hospital within 28 days.

### 3.3 Unadjusted association between baseline variables and 28-day mortality

Univariate Cox proportional hazards regression analysis was used to identify the key factors affecting the 28-day ICU and hospital mortality (Supplementary Table 1). The results demonstrated that age was significantly associated with both ICU mortality (HR 1.01 [95%CI 1.01−1.02], *P* < 0.0001) and hospital mortality (HR 1.02 [95%CI 1.01–1.02], *P* < 0.0001), showing an increasing risk with increasing age. BMI was also related to the mortality, with a HR of 0.99 (95%CI 0.98–1.00) for ICU mortality (*P* = 0.0240) and 0.99 (95%CI 0.98–1.00) for hospital mortality (*P* = 0.0096). Regarding gender, there was no significant difference in mortality risk between males and females for both ICU (HR 1.02 [95%CI 0.88 - 1.18], *P* = 0.8031) and hospital mortality (HR 0.99 [95%CI 0.88–1.11], *P* = 0.8286) . Among different ethnicity, African - Americans had a significant association with hospital mortality (HR 0.81 [95%CI 0.68−0.97], *P* = 0.0208), while other ethnic groups showed no significant impact on ICU mortality. The SOFA score was highly correlated with both ICU mortality (HR 1.27 [95%CI 1.24–1.29], *P* < 0.0001) and hospital mortality (HR 1.28 [95%CI 1.26–1.30], *P* < 0.0001), indicating an increased risk with a higher score. In laboratory tests, various parameters such as BUN, serum calcium, GLU, HGB, TC, HDL, RBC, RDW and WBC were significantly associated with mortality. For comorbidities, hepatic failure, lymphoma, metastatic cancer, immunosuppression, and cirrhosis were significantly associated with both ICU mortality and hospital mortality, while COPD, CHF, and AMI had different degrees of associations with hospital mortality but not significant for ICU mortality in some cases.

### 3.4 Adjusted association between TyG index and 28-day mortality

We employed three Cox proportional hazards regression models to evaluate the impact of the TyG index on 28-day all-cause mortality in critically ill patients (Table 2). The results revealed a significant association between the TyG index and ICU mortality, observed in both the unadjusted model (HR, 1.22 [95%CI 1.12–1.33], *P* < 0.0001) and the fully adjusted model (HR, 1.31 [95%CI 1.11–1.55], *P* = 0.0018). Furthermore, in the unadjusted model, the TyG index was also linked to hospital mortality (HR, 1.22 [95%CI 1.14–1.31], *P* < 0.0001), with this association persisting in the fully adjusted model (HR, 1.37 [95%CI 1.20–1.55], *P* < 0.0001). In the fully adjusted model, the risk of ICU mortality for the T2 and T3 groups of the TyG index was greater than that of the T1 group, demonstrating an increasing trend with rising TyG index (T1 vs. T2: HR, 1.32 [95%CI 0.99–1.77]; T3: HR, 1.73 [95%CI 1.26–2.35]; trend test *P* = 0.0005). Comparable results were observed in the Cox proportional hazards analysis of the TyG index and hospital mortality (T1 vs. T2: HR, 1.38 [95%CI 1.11–1.72]; T3: HR, 1.76 [95%CI 1.38–2.23]; trend test *P* < 0.0001).

**TABLE 2:** Relationship between TyG index and 28-day mortality in different models.

Model	Exposure	ICU mortality HR (95%CI) *P*-value	Hospital mortality HR (95%CI) *P*-value
**Model I**	**TyG index** **as continuous**	1.22 (1.12, 1.33) < 0.0001	1.22 (1.14, 1.31) < 0.0001
	T1	Ref	Ref
	T2	1.26 (1.03, 1.54) 0.0252	1.25 (1.07, 1.46) 0.0040
	T3	1.63 (1.35, 1.97) < 0.0001	1.60 (1.39, 1.85) < 0.0001
	P for trend	< 0.0001	<0.0001
**Model II**	**TyG index** **as continuous**	1.31 (1.20, 1.44) < 0.0001	1.35 (1.25, 1.45) < 0.0001
	T1	Ref	Ref
	T2	1.28 (1.04, 1.57) 0.0181	1.32 (1.13, 1.53) 0.0005
	T3	1.82 (1.50, 2.20) < 0.0001	1.86 (1.60, 2.16) < 0.0001
	P for trend	< 0.0001	<0.0001
**Model III**	**TyG index** **as continuous**	1.31 (1.11, 1.55) 0.0018	1.37 (1.20, 1.55) < 0.0001
	T1	Ref	Ref
	T2	1.32 (0.99, 1.77) 0.0628	1.38 (1.11, 1.72) 0.0034
	T3	1.72 (1.26, 2.35) 0.0006	1.76 (1.38, 2.23) < 0.0001
	P for trend	0.0005	< 0.0001

Model I: we did not account for additional variables. Model II: we adjusted gender, age, ethnicity. Model III: we adjusted gender, age, ethnicity, BMI, SOFA score, BUN, serum calcium, TC, HDL, RBC, HBG, WBC, RDW, hepatic failure, immunosuppression and cirrhosis. HR, Hazard Ratios; CI, confidence; Ref, reference.

### 3.5 Sensitivity analysis

Next, we showed the sensitivity analysis (Supplementary Table 2). In Model I, the analysis focused on Caucasian patients (*N* = 10,648). After adjusting for confounding variables, the TyG index was positively associated with the risk of 28-day all-cause mortality in the ICU, yielding a hazard ratio (HR) of 1.47 (95%CI 1.21–1.79, *P* = 0.0001). A similar association was observed between the TyG index and the risk of 28-day all-cause mortality in the hospital, with an HR of 1.50 (95%CI 1.30–1.74, *P* < 0.0001).

Model II examined patients aged ≥ 60 years (*N* = 8,563). In this model, the TyG index was positively associated with the risk of 28-day all-cause mortality in the ICU, with an HR of 1.35 (95%CI 1.11–1.66, *P* = 0.0035). The TyG index also demonstrated a positive correlation with the risk of 28-day all-cause mortality in the hospital, with an HR of 1.45 (95%CI 1.25–1.69, *P* < 0.0001).

In Model III, a generalized additive model (GAM) was employed, incorporating smooth terms for multiple variables. The TyG index was positively associated with the risk of 28-day all-cause mortality in the ICU, with an HR of 1.28 (95%CI 1.04–1.58, *P* = 0.0182), and it was also positively correlated with the risk of 28-day all-cause mortality in the hospital, with an HR of 1.38 (95%CI 1.17–1.62, *P* = 0.0002). The results of the sensitivity analyses underscore the robustness of our findings. Additionally, we calculated E-values to assess sensitivity to unmeasured confounding factors.

### 3.6 Identification of nonlinear relationship

**FIGURE 3:**
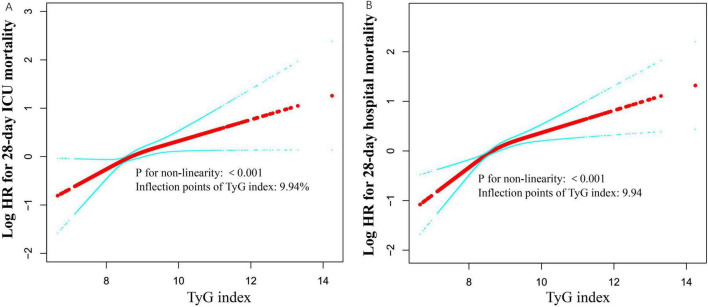
Associations between TyG index and 28-day mortality in critically ill patients. A threshold, nonlinear association between TyG index and 28-day mortality was identified using a generalized additive model (GAM). The solid red line represents the smooth curve fitted between the variables. The blue bands represent the 95% confidence interval (CI) from the fitted model **(A,B)**. The analysis was adjusted for gender, age, ethnicity, BMI, SOFA score, BUN, serum calcium, TC, HDL, RBC, HBG, WBC, RDW, hepatic failure, immunosuppression and cirrhosis.

Initially, we observed a nonlinear dose-response relationship between the TyG index and all-cause mortality in critically ill patients (Figure 3 and Table 3). When the TyG index is below 9.94, each 1-unit increase corresponds to a 49% increase in the adjusted risk of ICU mortality, with a HR of 1.49 (95%CI 1.20–1.85, *P* = 0.0018). Similarly, the adjusted risk of hospital mortality increases by 60%, with an HR of 1.60 (95%CI 1.35–1.89, *P* < 0.0001), indicating a significant elevation in mortality risk. Conversely, when the TyG index is equal to or greater than 9.94, the HR for ICU mortality is 0.93 (95%CI 0.61–1.41, *P* = 0.7220), and the HR for hospital mortality is 0.90 (95%CI 0.64–1.27, *P* = 0.5510), suggesting that the risk of mortality does not significantly increase with rising TyG index.

**TABLE 3:** Threshold effect analysis of the RDW and mortality.

Models	ICU mortality		Hospital mortality	
	**HR (95%CI)**	***P*-value**	**HR (95%CI)**	***P*-value**
**Model I**				
One line effect	1.31 (1.11, 1.55)	0.0018	1.37 (1.20, 1.55)	<0.0001
**Model II**				
Turning point (K)	9.94		9.94	
Weight change < K	1.49 (1.20, 1.85)	0.0003	1.60 (1.35, 1.89)	<0.0001
Weight change ≥ K	0.93 (0.61, 1.41)	0.7220	0.90 (0.64, 1.27)	0.5510
*P*-value for LRT test*	0.049		0.003	

Data were presented as HR (95%CI) *P*-value; Model I, linear analysis; Model II, non-linear analysis. Adjusted for gender, age, ethnicity, BMI, SOFA score, BUN, serum calcium, TC, HDL, RBC, HBG, WBC, RDW, hepatic failure, immunosuppression and cirrhosis. LRT logarithm likelihood ratio test.

*P < 0.05 indicates that model II is significantly different from Model I.

### 3.7 Results of subgroup analyses

**FIGURE 4:**
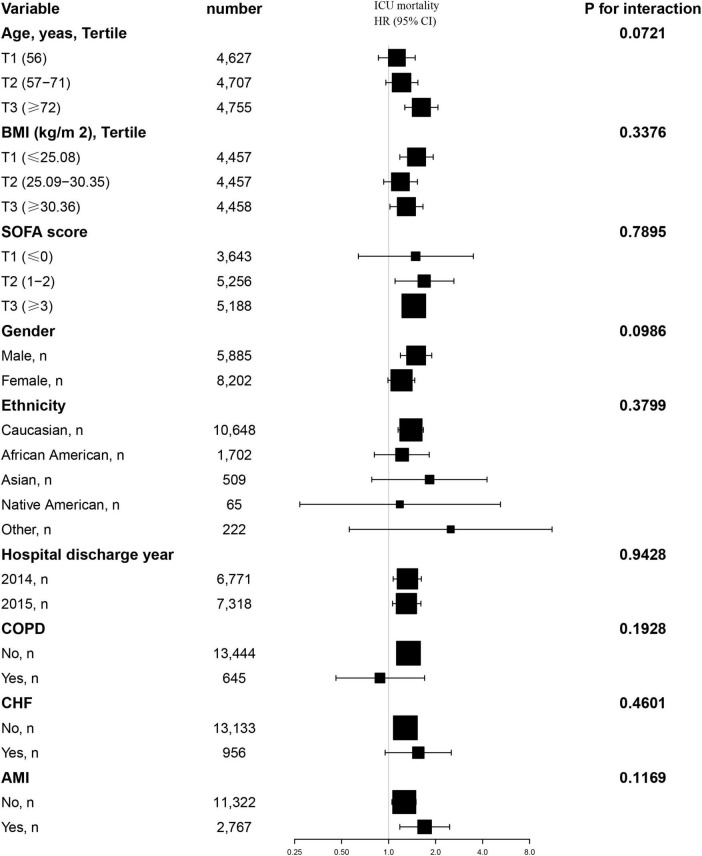
Effect size of TyG index on 28-day ICU mortality in prespecified and exploratory subgroups. Above model adjusted for gender, age, ethnicity, BMI, SOFA score, BUN, serum calcium, TC, HDL, RBC, HBG, WBC, RDW, hepatic failure, immunosuppression and cirrhosis. In each case, the model is not adjusted for the stratification variable when the stratification variable was a categorical variable.

**FIGURE 5:**
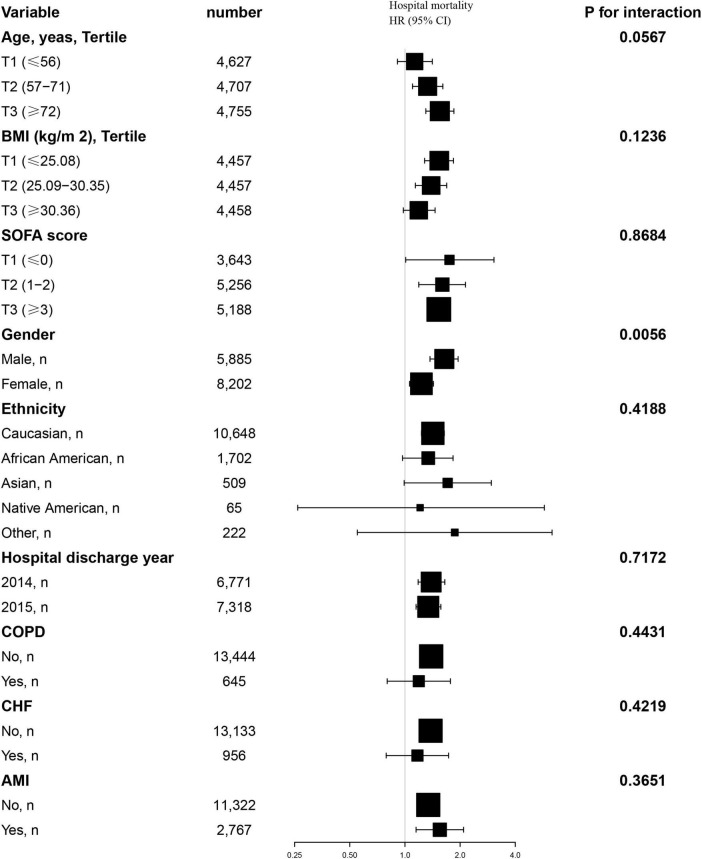
Effect size of TyG index on 28-day hospital mortality in prespecified and exploratory subgroups. Above model adjusted for gender, age, ethnicity, BMI, SOFA score, BUN, serum calcium, TC, HDL, RBC, HBG, WBC, RDW, hepatic failure, immunosuppression and cirrhosis. In each case, the model is not adjusted for the stratification variable when the stratification variable was a categorical variable.

Additionally, we conducted a stratified analysis to examine the relationship between the TyG index and all-cause mortality, considering potential modifying factors such as sex, age, BMI, SOFA score, ethnicity, hospital discharge year, COPD, CHF and AMI (Figures 4, 5). Our findings indicated that these factors did not significantly influence the association between the TyG index and the risk of 28-day all-cause mortality in the ICU, as all interaction *P*-values were greater than 0.05. Consistent results were observed in the stratified analysis of the TyG index and hospital mortality, with all interaction *P*-values also exceeding 0.05.

## 4 Discussion

We extracted information from the eICU database concerning 14,089 adult ICU patients to assess the association between the TyG index and clinical outcomes in non-diabetic critically ill patients in the United States. Our study found that the TyG index was significantly positively correlated with both the 28-day mortality rate in the ICU and the 28-day hospital mortality rate. This association remained significant after adjusting for various clinical and laboratory variables. Notably, we also identified a nonlinear saturation effect between the TyG index and 28-day all-cause mortality, with a saturation point at 9.94. Therefore, the TyG index may represent an important independent risk factor for non-diabetic critically ill patients and could serve as a valuable tool for informing clinical decision-making.

The mortality rate among critically ill patients in the Intensive Care Unit (ICU) is generally high. In the United States, there are approximately 4 million ICU admissions each year, with related data indicating that the mortality rate in this area ranges from 8 to 19%. Further analysis reveals that in some developed regions, the ICU mortality rate is relatively low; for example, it is 9.3% in North America and 10.3% in Oceania, while Europe exhibits a higher rate of 18.7%. In developed Asian regions, the ICU mortality rate is 13.7%. In contrast, South America shows a significant increase in ICU mortality, reaching 21.7%, and the situation is even more severe in the Middle East, where the mortality rate can be as high as 26.2%. In some underdeveloped regions of Africa, the ICU mortality rate may even reach 53.6% (27). In our study, we observed that within 28 days, 730 patients (5.18% of the total) died in the ICU, while the total number of patients who died in the hospital was 1,178, accounting for 8.36%. Research indicates that the length of stay in the ICU is correlated with mortality; the longer the stay, the higher the risk of death (28). In this study, the average length of stay for the observed subjects was 6.51 days. Additionally, this study calculated mortality data only within 28 days, which may partially explain why the observed mortality rate is lower than the average level in the United States. Overall, the mortality rate among critically ill non-diabetic patients remains high, particularly in underdeveloped areas, underscoring the urgent need for a cost-effective, rapid, and effective predictive indicator to reduce the mortality risk of critically ill patients.

Insulin resistance is frequently observed in critically ill patients and correlates with the severity of illness (29, 30). The TyG index has gained widespread acceptance as a marker of IR. Recent studies have demonstrated a robust association between the TyG index and critically ill patients. Specifically, the TyG index is significantly linked to all-cause mortality in patients with ischemic stroke, as well as those with hemorrhagic stroke (13, 31). In a retrospective cohort study involving 1,618 patients with severe coronary artery disease, Zhang et al. (32) reported that the TyG index was significantly associated with ICU mortality, yielding a HR of 1.50. Additionally, Zheng et al. (14) highlighted in a cross-sectional study of 1,257 patients with sepsis that a higher TyG index was associated with an increased risk of in-hospital mortality among critically ill patients. Notably, the proportion of non-diabetic patients in the critically ill population is significantly higher than that of diabetic patients. This study indicates that approximately 50% of critically ill patients in the ICU experience hyperglycemia within the first 48 h of admission. Stress-induced hyperglycemia is regarded as a marker of disease severity, and the extent of hyperglycemia is strongly correlated with short-term mortality, particularly in patients without a prior history of diabetes (33, 34). The TyG index, as a surrogate marker of IR, reflects underlying metabolic dysregulation, including impaired insulin signaling in target tissues and compensatory hyperinsulinemia, which are key drivers of adverse outcomes in critically ill patients (35, 36). In non-diabetic critically ill patients, IR and its associated hyperinsulinemia may exacerbate systemic inflammation and oxidative stress, promote endothelial dysfunction, and disrupt lipid metabolism, all of which contribute to organ damage and increased mortality (37, 38). Several studies have demonstrated that hyperinsulinemia can activate pro-inflammatory pathways, leading to elevated levels of cytokines such as IL-6 and TNF-α, which further impair vascular function and promote atherosclerosis (39). In addition, IR-induced lipolysis increases circulating free fatty acids (FFAs), which can induce lipotoxicity and mitochondrial dysfunction, particularly in the liver and cardiovascular system (40). Furthermore, IR impairs the PI3K/Akt signaling pathway, reducing nitric oxide (NO) synthesis and leading to endothelial dysfunction, which exacerbates thrombosis and vascular complications (41, 42). These mechanisms collectively emphasize the critical role of IR and hyperinsulinemia in worsening outcomes in non-diabetic critically ill patients, highlighting the need for clinical monitoring and management of these metabolic dysregulations. We reviewed the literature and found that there are few studies examining the outcomes of non-diabetic critically ill patients concerning the TyG index. Existing studies indicate that the HR for the TyG index in patients without diabetes is significantly associated with a value of 2.83 (1.95–4.12), suggesting that the TyG index can more effectively identify mortality risk in these patients. This finding implies that utilizing the TyG index as a monitoring tool may assist physicians in better assessing and managing non-diabetic critically ill patients in the ICU (8). However, the number of non-diabetic patients included in this study was only 2,198, which is insufficient for robust conclusions. Consequently, large-scale cohort studies are necessary to further validate the association between the TyG index and mortality risk in non-diabetic critically ill patients.

In comparison to other studies, our research included a total of 14,089 non-diabetic patients, which is significantly larger than the sample sizes reported in previous research (8, 31, 43). We observed that as the TyG index quartiles increased, both the 28-day ICU mortality rate and hospital mortality rate exhibited a significant increase. Furthermore, our analysis utilizing the Cox proportional hazards regression model indicated a significant positive association between the TyG index and both ICU and hospital mortality, consistent across both unadjusted and fully adjusted models. This finding aligns with the majority of studies (8, 31). However, the effect sizes identified in our study differ slightly from those reported in other research, potentially due to variations in the adjusted covariates. Similar to most studies, we also identified a nonlinear relationship between the TyG index and 28-day all-cause mortality (31). Notably, we observed a saturation effect regarding the TyG index and ICU or hospital mortality; specifically, when the TyG index is below 9.94, the ICU or hospital mortality rates significantly increase with rising TyG index values. Conversely, when the TyG index exceeds 9.94, the ICU or hospital mortality rates demonstrate minimal change despite increasing TyG index values. This finding is consistent with the study conducted by Zhang et al. (43), although their saturation point occurs at a lower TyG index, which may be attributed to their study population comprising entirely of patients with severe sepsis, who exhibited a higher mortality rate than those in our study.

Our study presents several significant advantages. First, our study population comprised 14,089 non-diabetic critically ill patients and utilized a multicenter retrospective cohort study design. This approach enhances the representativeness and applicability of the study results compared to studies with smaller sample sizes or case-control designs. Second, we not only validated the positive correlation between the TyG index and 28-day all-cause mortality in the ICU, but also confirmed a similar relationship with 28-day all-cause mortality in the hospital setting. In studies targeting this population, we observed a nonlinear saturation effect between the TyG index and 28-day all-cause mortality, identifying a critical threshold of 9.94. This finding enables clinicians to identify patients with a high TyG index, facilitating timely attention to individuals at greater risk of mortality, along with effective intervention and monitoring. Third, we conducted a comprehensive sensitivity analysis to verify the stability of the TyG index as a predictor of mortality risk, thereby enhancing the reliability of our results. Fourth, our study included subgroup analyses and interaction assessments, which deepen our understanding of the performance of the TyG index across different patient populations. Finally, our analysis thoroughly considered various potential confounding factors, including sex, age, ethnicity, BMI, SOFA score, BUN, serum calcium, total cholesterol, HDL, red blood cells, HGB, white blood cells, red cell distribution width, liver failure, immunosuppression, and cirrhosis. Additionally, we employed E-values to assess sensitivity to unmeasured confounding factors, thereby increasing the rigor of the study, in contrast to many previous studies that may have yielded biased outcomes due to insufficient adjustment for these confounding factors.

This study does have several notable limitations. First, the retrospective study design may have introduced selection and information biases, potentially affecting the interpretation of the results and the reliability of the conclusions, particularly regarding patient selection and data collection. Second, while we implemented measures to control for various confounding factors, there may still be unidentified variables. To address this, we conducted E-value sensitivity analyses to evaluate the potential impact of unmeasured confounding factors, and the results suggested that these factors are unlikely to fully account for the treatment effects. Third, although the study data were sourced from over 200 hospitals in the United States, the generalizability of the results may be limited and may not broadly apply to ICU patients in other countries or regions. Fourth, our understanding of the relevant mechanisms remains incomplete, and future research should prioritize exploring these physiological mechanisms. Finally, the study data were derived from the eICU database, which reflects the characteristics of a specific population, region, or time period, indicating that the results may not be applicable to all other populations or contexts of interest to researchers, thereby somewhat constraining the generalizability and external validity of the study findings.

## 5 Conclusion

This study conducted a retrospective analysis of 14,089 non-diabetic critically ill patients using the eICU database. The results showed a significant positive correlation between the TyG index and 28-day all-cause mortality in both ICU and hospital settings. Additionally, the study identified a nonlinear saturation effect between the TyG index and mortality, with a saturation point at 9.94. Below this threshold, each unit increase in the TyG index was significantly associated with an elevated mortality risk, while above this value, the mortality risk did not significantly increase.

## Data Availability

The original contributions presented in this study are included in this article/Supplementary material, further inquiries can be directed to the corresponding authors.

## Footnotes

1
www.empowerstats.com

2
http://www.r-project.org
