# Supplementary material for: Triglyceride-glucose index and prognosis in non-diabetic critically ill patients: data from the eICU database
Source: Front Med (Lausanne). 2025 Apr 8;12:1558968. doi: 10.3389/fmed.2025.1558968 (PMC12011771; doi:10.3389/fmed.2025.1558968)
Supplement: Supplementary file 1 [file Data_Sheet_1.docx]

**Triglyceride-Glucose Index and Prognosis in Non-Diabetic critically ill Patients: data from the eICU Database**

Supplementary Material

**Xi Li^1†^, Qiujin Lin^2†^, Dewen Zhang^3†^, Zhenhua Huang^4^, Jinshi Yu^1^，Jiaqi Zhao^1^, Wenzhou Li^5*^, Wei Liu^6*^**

^1^Pharmacy Department, Shenzhen Qianhai Shekou Free Trade Zone Hospital, Shenzhen, China

^2^Department of Critical Care Medicine, Pengpai Memorial Hospital, Shanwei, China

^3^Department of Pharmacy, Pengpai Memorial Hospital, Shanwei, China

^4^Department of Emergency Medicine, the First Affiliated Hospital of Shenzhen University &Shenzhen Second People’s Hospital, Shenzhen, China

^5^Shenzhen Baoan Women's and Children's Hospital, Shenzhen, China

^6^Department of Emergency Medicine, The Huangpu People’s Hospital of Zhongshan, Zhongshan, China

**Supplementary Table 1** Factors influencing risk of ICU and hospital mortality analyzed by univariate Cox proportional hazards regression analysis

| **Variable** | **Statistics** | **ICU mortality**  **HR (95% CI) P-value** | **Hospital mortality**  **HR (95% CI) P-value** |
| --- | --- | --- | --- |
| Age（year） | 63.47 ± 15.89 | 1.01 (1.01, 1.02) <0.0001 | 1.02 (1.01, 1.02) <0.0001 |
| BMI (kg/m^2^) | 28.77 ± 7.56 | 0.99 (0.98, 1.00) 0.0240 | 0.99 (0.98, 1.00) 0.0096 |
| **Gender** |  |  |  |
| Male, n (%) | 5885 (41.78%) | 1.0 | 1.0 |
| Female, n (%) | 8202 (58.22%) | 1.02 (0.88, 1.18) 0.8031 | 0.99 (0.88, 1.11) 0.8286 |
| **Ethnicity** |  |  |  |
| Caucasian, n (%) | 10648 (76.39%) | 1.0 | 1.0 |
| African-American, n (%) | 1702 (12.21%) | 0.94 (0.75, 1.16) 0.5407 | 0.81 (0.68, 0.97) 0.0208 |
| Hispanic, n (%) | 793 (5.69%) | 1.00 (0.73, 1.36) 0.9807 | 0.76 (0.57, 1.01) 0.0548 |
| Asian, n (%) | 509 (3.65%) | 0.87 (0.59, 1.31) 0.5139 | 0.97 (0.73, 1.30) 0.8484 |
| Native American, n (%) | 65 (0.47%) | 1.50 (0.62, 3.62) 0.3654 | 1.51 (0.72, 3.18) 0.2767 |
| Unknown, n (%) | 222 (1.59%) | 0.85 (0.47, 1.54) 0.5833 | 0.93 (0.59, 1.47) 0.7566 |
| SOFA score, median (IQR) | 1.00 (0.00-4.00) | 1.27 (1.24, 1.29) <0.0001 | 1.28 (1.26, 1.30) <0.0001 |
| **Laboratory tests** |  |  |  |
| BUN, median (IQR), (mmol/L) | 16.00 (12.00-24.00) | 1.01 (1.01, 1.01) <0.0001 | 1.01 (1.01, 1.01) <0.0001 |
| Serum calcium | 8.48 ± 0.74 | 0.83 (0.76, 0.90) <0.0001 | 0.81 (0.76, 0.87) <0.0001 |
| GLU, median (IQR), (mg/dl) | 118.0 (100.0-145.0) | 1.00 (1.00, 1.00) <0.0001 | 1.00 (1.00, 1.00) <0.0001 |
| HGB, (g/L) | 12.24 ± 2.27 | 0.96 (0.93, 0.99) 0.0032 | 0.95 (0.93, 0.97) <0.0001 |
| PLT, (×10^9^/L) | 212.43 ± 85.88 | 1.00 (1.00, 1.00) 0.1788 | 1.00 (1.00, 1.00) 0.0151 |
| TC, (mg/dl) | 156.20 ± 49.40 | 0.99 (0.99, 1.00) <0.0001 | 0.99 (0.99, 1.00) <0.0001 |
| TG, median (IQR), (mg/dl) | 104.0 (74.0-154.0) | 1.00 (1.00, 1.00) 0.1225 | 1.00 (1.00, 1.00) 0.1099 |
| HDL, (mg/dl) | 42.69 ± 16.51 | 0.99 (0.98, 1.00) 0.0003 | 0.99 (0.99, 1.00) 0.0010 |
| RBC. (×10/^12^L) | 4.08 ± 0.74 | 0.89 (0.81, 0.97) 0.0094 | 0.87 (0.80, 0.93) 0.0001 |
| RDW (%) | 14.57 ± 2.13 | 1.09 (1.06, 1.12) <0.0001 | 1.11 (1.09, 1.13) <0.0001 |
| WBC, (×10^9^/L) | 11.26 ± 6.88 | 1.02 (1.01, 1.02) <0.0001 | 1.02 (1.01, 1.02) <0.0001 |
| **Comorbidities** |  |  |  |
| Hepatic failure, n (%) | 91 (0.70%) | 1.99 (1.12, 3.51) 0.0185 | 1.78 (1.12, 2.84) 0.0151 |
| Lymphoma, n (%) | 28 (0.22%) | 2.03 (0.51, 8.13) 0.3185 | 2.22 (0.92, 5.33) 0.0760 |
| Metastatic cancer, n (%) | 167 (1.29%) | 1.51 (0.93, 2.44) 0.0928 | 2.08 (1.50, 2.88) <0.0001 |
| Leukemia, n (%) | 67 (0.52%) | 1.42 (0.71, 2.85) 0.3249 | 1.28 (0.67, 2.47) 0.4553 |
| Immunosuppression, n (%) | 236 (1.82%) | 2.01 (1.41, 2.86) 0.0001 | 1.87 (1.40, 2.49) <0.0001 |
| Cirrhosis, n (%) | 114 (0.88%) | 2.00 (1.23, 3.23) 0.0049 | 1.99 (1.36, 2.92) 0.0004 |
| COPD, n (%) | 645 (4.58%) | 0.89 (0.65, 1.22) 0.4685 | 1.09 (0.86, 1.38) 0.4770 |
| CHF, n (%) | 956 (6.79%) | 0.98 (0.76, 1.27) 0.8958 | 0.97 (0.79, 1.19) 0.7831 |
| AMI, n (%) | 2767 (19.64%) | 0.94 (0.76, 1.18) 0.6173 | 0.79 (0.66, 0.95) 0.0135 |

CI, confidence interval; OR, odds ratio.

**Supplementary Table 2.** Relationship between TyG index and 28-day mortality in different sensitivity analyses.

| **Model** | **Exposure** | **ICU mortality**  **HR (95% CI) P-value** | **Hospital mortality**  **HR (95% CI) P-value** |
| --- | --- | --- | --- |
| **Model I** | **TyG index as continuous** | 1.47 (1.21, 1.79) 0.0001 | 1.50 (1.30, 1.74) <0.0001 |
|  | T1 | Ref | Ref |
|  | T2 | 1.49 (1.06, 2.09) 0.0222 | 1.46 (1.14, 1.87) 0.0026 |
|  | T3 | 2.06 (1.44, 2.94) <0.0001 | 1.91 (1.46, 2.49) <0.0001 |
|  | P for trend | <0.0001 | 1.38 (1.20, 1.57) <0.0001 |
| **Model II** | **TyG index as continuous** | 1.35 (1.11, 1.66) 0.0035 | 1.45 (1.25, 1.69) <0.0001 |
|  | T1 | Ref | Ref |
|  | T2 | 1.67 (1.20, 2.33) 0.0026 | 1.52 (1.20, 1.94) 0.0007 |
|  | T3 | 1.96 (1.37, 2.82) 0.0003 | 1.86 (1.42, 2.44) <0.0001 |
|  | P for trend | <0.0001 | <0.0001 |
| **Model III** | **TyG index as continuous** | 1.28 (1.04, 1.58) 0.0182 | 1.38 (1.17, 1.62) 0.0002 |
|  | T1 | Ref | Ref |
|  | T2 | 1.28 (0.87, 1.89) 0.2041 | 1.26 (0.95, 1.69) 0.1128 |
|  | T3 | 1.64 (1.11, 2.42) 0.0134 | 1.71 (1.26, 2.31) 0.0005 |
|  | P for trend | 0.0113 | 0.0004 |

Model I was a sensitivity analysis performed with Caucasian patients (N= 10648). we adjusted gender, age, ethnicity, BMI, SOFA score, BUN, serum calcium, TC, HDL, RBC, HBG, WBC, RDW, hepatic failure, immunosuppression and cirrhosis.

Model II was a sensitivity analysis performed patients’ age≥60 years (N= 8563). we adjusted we adjusted gender, age, ethnicity, BMI, SOFA score, BUN, serum calcium, TC, HDL, RBC, HBG, WBC, RDW, hepatic failure, immunosuppression and cirrhosis.

Model III was a sensitivity analysis performed patients’ BMI≥25 kg/m^2^ (N= 8563). we adjusted we adjusted gender, age, ethnicity, BMI, SOFA score, BUN, serum calcium, TC, HDL, RBC, HBG, WBC, RDW, hepatic failure, immunosuppression and cirrhosis.


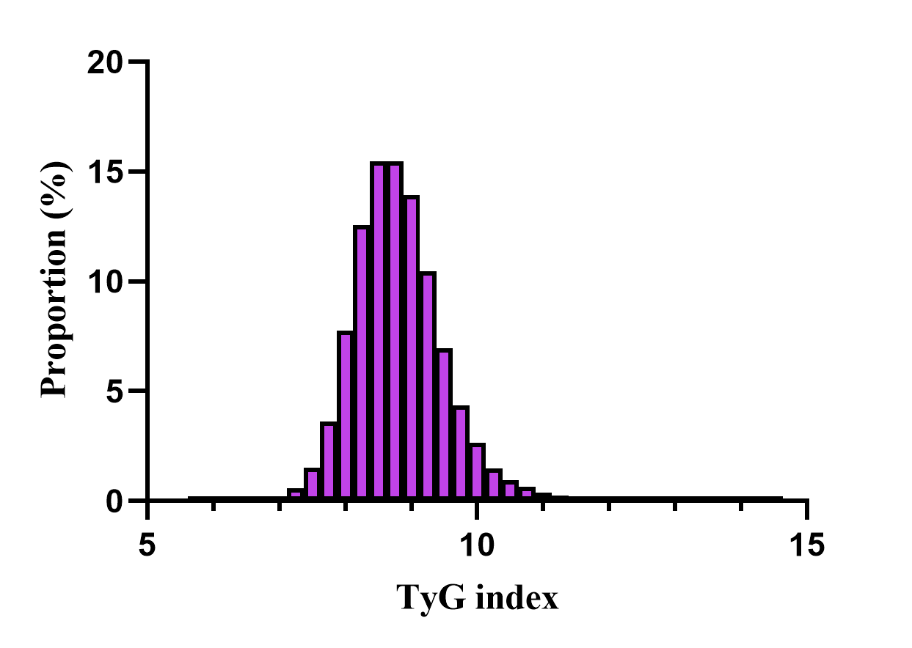


**Supplementary Figure 1.** Distribution of TyG index. It presented a normal distribution, ranging from 5.70 to 14.44, with a mean of 8.83.


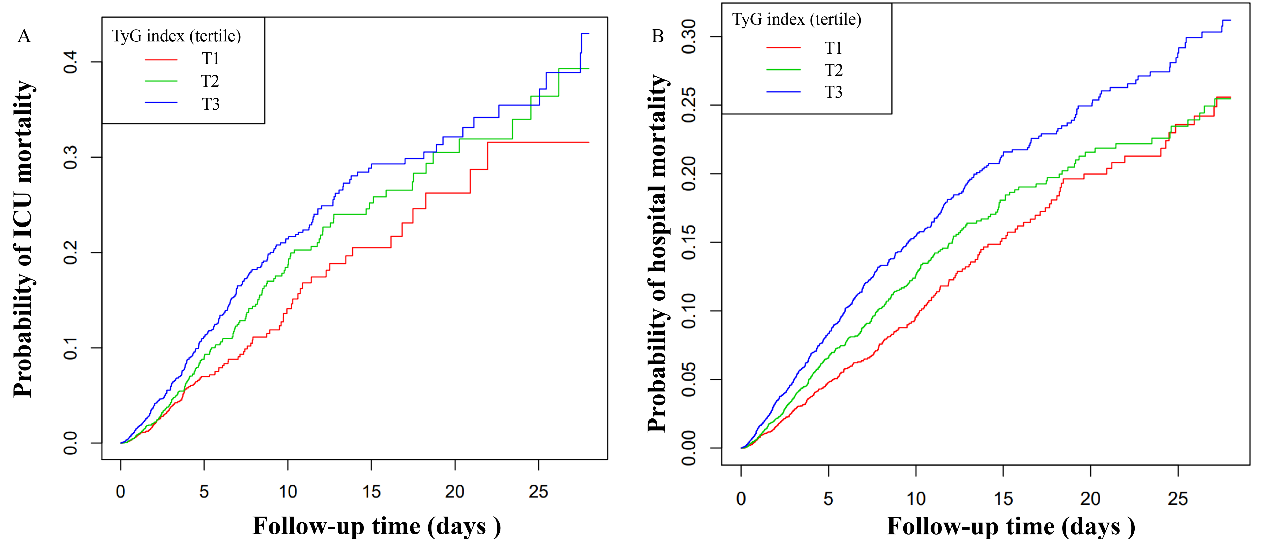


**Supplementary Figure 2.** The Kaplan–Meier curves for 28-day ICU (A) and 28-day hospital mortality (B). The probability of 28-day ICU and hospital mortality increased progressively as the tertiles of the TyG index rose.
